# Induction chemotherapy followed by alternating chemo-radiotherapy in stage IV undifferentiated nasopharyngeal carcinoma

**DOI:** 10.1054/bjoc.2000.1485

**Published:** 2000-11-22

**Authors:** M Benasso, G Sanguineti, M D'Amico, R Corvò, I Ricci, G Numico, D Guarneri, V Vitale, E Pallestrini, A Santelli, R Rosso

**Affiliations:** Department of Medical Oncology I, Radiation Oncology, Istituto Nazionale per la Ricerca sul Cancro, Genova; Department of Medical Oncology, Ospedale S. Croce e Carle, Cuneo; Department of Medical Oncology, Ospedale Sanremo; Department of Otolaryngology, Ospedale S. Martino, Genova

**Keywords:** nasopharyngeal carcinoma, chemo-radiotherapy, combined treatments

## Abstract

In locally advanced undifferentiated nasopharyngeal carcinoma (UNPC), concomitant chemo-radiotherapy is the only strategy that gave better results over radiation alone in a phase III trial. Adding effective chemotherapy to a concomitant chemo-radiotherapy programme may be a way to improve the results further. 30 patients with previously untreated T4 and/or N2–3 undifferentiated nasopharyngeal carcinoma were consecutively enrolled and initially treated with 3 courses of epidoxorubicin, 90 mg/m2, day 1 and cisplatin, 40 mg/m2, days 1 and 2, every 3 weeks. After a radiological and clinical response assessment patients underwent 3 courses of cisplatin, 20 mg/m2/day, days 1–4 and fluorouracil, 200 mg/m2/day, days 1–4, i.v. bolus, (weeks 1, 4, 7) alternated to 3 courses of radiation (week 2–3, 5–6, 8–9–10), with a single daily fractionation, up to 70 Gy. WHO histology was type 2 in 30% and type 3 in 70% of the patients. 57% had T4 and 77% N2–3 disease. All the patients are evaluable for toxicity and response. All but one received 3 courses of induction chemotherapy. Toxicity was mild to moderate in any case. At the end of the induction phase 10% of CRs, 83.3% of PRs and 6.7% of SD were recorded. All the patients but one had the planned number of chemotherapy courses in the alternating phase and all received the planned radiation dose. One patient out of 3 developed grade III–IV mucositis. Haematological toxicity was generally mild to moderate. At the final response evaluation 86.7% of CRs and 13.3% of PRs were observed. At a median follow-up of 31 months, 13.3% of patients had a loco-regional progression and 20% developed distant metastases. The 3-year actuarial progression-free survival and overall survival rates were 64% and 83%. Induction chemotherapy followed by alternating chemo-radiotherapy is feasible and patients' compliance optimal. This approach showed a very promising activity on locally advanced UNPC and merits to be investigated in phase III studies. © 2000 Cancer Research Campaign http://www.bjcancer.com


*British Journal of Cancer* (2000) **83**(11), 1437–1442
© 2000 Cancer Research Campaign
doi: 10.1054/ bjoc.2000.1485, available online at http://www.idealibrary.com on IDEAL®

http://www.bjcancer.com

### Induction chemotherapy followed by alternating chemo-radiotherapy in stage IV undifferentiated nasopharyngeal carcinoma

**M Benasso[1], G Sanguineti[2], M D'Amico[1], R Corvò[2], I Ricci[1], G Numico[3], D Guarneri, V Vitale[2], E Pallestrini[5], A Santelli[5] and R Rosso[1]**

[1]Department of Medical Oncology I and [2]Radiation Oncology, Istituto Nazionale per la Ricerca sul Cancro, Genova; [3]Department of Medical Oncology, Ospedale S. Croce e Carle, Cuneo; [4]Department of Medical Oncology, Ospedale Sanremo; [5]Department of Otolaryngology, Ospedale S. Martino, Genova

Summary In locally advanced undifferentiated nasopharyngeal carcinoma (UNPC), concomitant chemo-radiotherapy is the only strategy that gave better results over radiation alone in a phase III trial. Adding effective chemotherapy to a concomitant chemo-radiotherapy programme may be a way to improve the results further. 30 patients with previously untreated T4 and/or N2–3 undifferentiated nasopharyngeal carcinoma were consecutively enrolled and initially treated with 3 courses of epidoxorubicin, 90 mg/m2, day 1 and cisplatin, 40 mg/m2, days 1 and 2, every 3 weeks. After a radiological and clinical response assessment patients underwent 3 courses of cisplatin, 20 mg/m2/day, days 1–4 and fluorouracil, 200 mg/m2/day, days 1–4, i.v. bolus, (weeks 1, 4, 7) alternated to 3 courses of radiation (week 2–3, 5–6, 8–9–10), with a single daily fractionation, up to 70 Gy. WHO histology was type 2 in 30% and type 3 in 70% of the patients. 57% had T4 and 77% N2–3 disease. All the patients are evaluable for toxicity and response. All but one received 3 courses of induction chemotherapy. Toxicity was mild to moderate in any case. At the end of the induction phase 10% of CRs, 83.3% of PRs and 6.7% of SD were recorded. All the patients but one had the planned number of chemotherapy courses in the alternating phase and all received the planned radiation dose. One patient out of 3 developed grade III–IV mucositis. Haematological toxicity was generally mild to moderate. At the final response evaluation 86.7% of CRs and 13.3% of PRs were observed. At a median follow-up of 31 months, 13.3% of patients had a loco-regional progression and 20% developed distant metastases. The 3-year actuarial progression-free survival and overall survival rates were 64% and 83%. Induction chemotherapy followed by alternating chemo-radiotherapy is feasible and patients' compliance optimal. This approach showed a very promising activity on locally advanced UNPC and merits to be investigated in phase III studies. © 2000 Cancer Research Campaign http://www.bjcancer.com

**Keywords**: nasopharyngeal carcinoma; chemo-radiotherapy; combined treatments

Nasopharyngeal cancers (NPC) are generally considered all those tumours of epidermoid origin arising in the nasopharynx. They constitute over 90% of tumours of this anatomical site. Basing on the degree of differentiation, the World Health Organization (WHO) distinguished 3 different types of NPC: squamous cell keratinizing carcinoma (type 1), non-keratinizing carcinoma (type 2) and undifferentiated carcinoma (type 3). According to the Micheau classification (Micheau et al, 1981), NPC are divided into two categories: squamous cell carcinoma and undifferentiated carcinoma of nasopharyngeal type. Generally, the definition of 'undifferentiated nasopharyngeal carcinoma' (UNPC) is restricted to the type 2 and 3 of the WHO classification or to the undifferentiated carcinoma of nasopharyngeal type of the Micheau classification. To distinguish the UNPC from the other epithelial tumours of the nasopharynx is crucial since it widely differs from the others in terms of epidemiology, clinical behaviour, pattern of spread and sensitivity to radiation.

The incidence of UNPC is very low in Western countries (0.5–2/100 000/year) but it rises in the Mediterranean basin countries (8–12/100 000/year) and reaches the highest rates in the south of China (25–50/100 000/year) (Ho, 1978; Ho, 1982; Levine and Conelly, 1985, pp 13–34; Li, 1985). Radiotherapy is the historically accepted treatment for the UNPC. Unfortunately, most of the studies with the largest series of patients include both early and advanced stages, well differentiated and undifferentiated tumours so that it is difficult to have clear data on radiation efficacy in UNPC, stage by stage. Even with this limit, it is possible to say that while in early stages radiation can give satisfactory long term results (5-year survival: 75–95%) (Huang et al, 1985; Chatani et al, 1986; Dexing et al, 1988), in locally advanced disease (T4 and/or N2–3), despite an overall immediate local control rate even higher than 70%, the 5-year survival rate is 15–40% in most of the studies (Huang, 1980; Chu et al, 1984; Zhang et al, 1987; Qin et al, 1988; Lee et al, 1992). Control of primary tumour and prevention of distant dissemination are both major issues in locally advanced disease. Local recurrence occurs in 15 to 54% of cases at 5 years, mainly influenced by tumour stage and cranial nerve involvement, as well as histological type (Mesic et al, 1981; Shwaab, 1983, pp 88–89; Sanguineti et al, 1997). The distant metastases rate is 20 to 35% at 5 years and reaches 60 to 80% for patients with N3 disease (Cvitkovic, 1991; Lee et al, 1992; Sanguineti et al, 1997).

Experience with metastatic or recurring patients showed that UNPC is a tumour sensitive to chemotherapy, particularly cisplatin. Therefore, chemotherapy has been introduced also in patients with

*Received 25 February 2000*
*Revised 25 July 2000*
*Accepted 9 August 2000*

*Correspondence to:* M Benasso advanced locoregionally-confined disease in order to improve results over radiation alone. However, the role of chemotherapy in a front line treatment of locally advanced disease is not yet definitively assessed. In fact, at present, only one randomized trial showed a statistically significant benefit in survival adding chemotherapy to radiation (Al Sarraf et al, 1998).

Here we report data from a prospective phase II trial testing the feasibility and the activity in terms of complete response rates, progression-free survival and overall survival of a treatment programme including induction chemotherapy followed by an alternating chemo-radiotherapy regimen. The aim of this strategy was both to improve local control through a consolidated alternating regimen that already showed this capacity in squamous cell carcinoma (Merlano et al, 1992), and distant control through the administration of more cycles of chemotherapy given at cytotoxic doses even in the chemo-radiation phase. The induction regimen chosen included cisplatin and epirubicin because, although sufficient single agent phase II data is unavailable, the combination of these two drugs gave high response rates and acceptable toxicity (Licitra et al, 1994; Rahal et al, 1994; Oliveira et al, 1996; Chua et al, 1998).

### MATERIALS AND METHODS

### Selection of patients

Patients were elegible for the study if they had the following features: histologically proven type 2 or 3 nasopharyngeal carcinoma according to the WHO classification; T4 and/or N2–3 disease (stage IV) according to the UICC 1992 criteria; no previous treatments; no distant metastases or other malignancies; age less than 71; performance status less than 2 (ECOG scale) (Miller et al, 1981); no major abnormalities of liver, heart, lung and bone marrow function; informed consent.

Pretreatment evaluation included physical and ENT examination, computerized tomography of the skull base and neck, complete blood counts and blood chemistry profiles, chest X-ray, liver echography and bone scan examination.

### Treatment

The treatment protocol consisted of 3 courses of induction chemotherapy followed by an alternating chemo-radiotherapy programme. Each course of induction chemotherapy consisted of cisplatin, 40 mg/m$^2$/day, days 1 and 2, and epirubicin, 90 mg/m$^2$, day 1, every 3 weeks. The alternating programme generally started 4 weeks after the last induction course and consisted of 3 courses of cisplatin, 20 mg/m$^2$/day and fluorouracil 200 mg/m$^2$/day for 4 consecutive days (weeks 1, 4, 7) alternated to 3 split courses of radiation (weeks 2–3, 5–6, 8–9–10). Cisplatin was given during a 2-hour period of forced hydration. Fluorouracil was given as an intravenous bolus dose at the end of cisplatin hydration. Antiemetic therapy consisted of dexametasone, 8 mg i.v. and tropisetron, 3 mg i.v., before each chemotherapy administration.

In the induction phase patients recycled after 3 weeks if the neutrophils count was ≥ 1500 cells μl$^{-1}$ and the platelets counts was ≥ 90 000 cells μl$^{-1}$, otherwise one more week of recovery was planned. In case of grade IV neutropenia and/or thrombocytopenia, a 20% drugs reduction was done at the following course. In the alternating programme if the neutrophil count was < 1500 cells μl$^{-1}$ and the platelet count was < 90000 cells μl$^{-1}$ the chemotherapy course was delayed for one week but the patient continued to be treated with radiation in order to avoid breaks during the chemoradiation regimen. The routine administration of granulocyte colony-stimulating factors was not allowed; their use was considered in single cases in order to reduce the risk of neutropenic fever and to maintain the intervals between cycles within 4 weeks. In no case was chemotherapy delivered concomitantly with radiation. Temporary treatment interruptions were allowed only in cases of grade IV mucositis.

Radiotherapy consisted of 70 gray delivered as daily single doses of 2 gray, 5 times a week during weeks 2–3, 5–6 and 8–9–10. For the initial 50 gray, two lateral and parallel opposed photon (cobalt or 6 MV) customized fields were used to treat the primary site and upper-mid neck nodes. The dose was specified and calculated at midplane along the central axis for lateral opposed beams. 50 gray were prescribed to electively treat neck regions as well. The lower neck–supraclavicular regions were treated with an appositional, anteroposterior field, with the dose prescribed at the depth of 3 cm. Areas of initial disease on both tumour and/or node sites were boosted to 60 gray regardless of response to induction chemotherapy. Areas of residual primary and/or nodal, postinduction chemotherapy disease were treated to 70 gray. This was generally done by the usual shrinking field technique. During this phase, higher energy (15–18 MV photons) were used to treat the primary site. However, for 12 T4 patients, a conformal 3D approach with multiple co-planar fields was attempted. The dose to the spinal cord was to be limited to 50 gray.

### Evaluation of response and toxicity

The response to treatment was assessed at the end of induction chemotherapy and 2 months after the end of the alternating programme through an ENT evaluation and a computerized tomography of the skull base and neck. Complete response (CR) was defined as the complete disappearance of all lesions; partial response (PR) was defined as a 50% or more reduction of the sum of the products of the two longest diameters of all lesions. Treatment failure was indicated by stable disease (SD), defined as a decrease of less than 50% or an increase of less than 25%; disease progression (PD), defined as an increase of more than 25%; early death (ED), defined as any death occurring before the end of treatment for tumour and treatment due to unrelated causes.

Toxicities were evaluated according to the WHO scale (Miller et al, 1981) and recorded as the worst grade experienced by patients during the treatment.

Patients underwent a follow-up programme including a monthly physical examination (in the first year, then every 3 months), an ecography of the neck every 3 months (at least in the first year), a CT scan of the skull base and neck and a chest X-ray every 6 months and a bone scan examination every year.

### Progression-free survival and overall survival

Progression-free survival was computed from the time of treatment beginning until that of disease progression at any site including distant metastasis or second primary tumours. Patients who died without evidence of disease progression were considered to have had progressive disease at the time of death. Overall survival was computed from the time of treatment beginning until the time of the last follow-up or death. All patients were included in the above analyses.

*© 2000 Cancer Research Campaign*

Actuarial survival and progression-free survival analyses were carried out according to the Kaplan-Meier method (Kaplan and Meier, 1958).

### RESULTS

Between May 1995 and June 1999, 30 consecutive patients (18 males and 12 females) entered this phase II prospective study. The median age was 50 (range: 21–70) and the median performance status was 0 (range: 0–1). 9 patients (30%) had a WHO type 2 and 21 patients (70%) type 3 histology. Tumour and nodal extensions are listed in Table 1. 57 percent of the patients had T4 and 77% N2–3 disease; 33% of the patients had both T4 and N2–3 lesions.

All the patients completed the treatment programme and are fully evaluable for toxicity and response. The compliance to the treatment was good (Table 2). 28 patients (93%) received all the 6 courses of chemotherapy planned in the 2 phases of the treatment.

### Induction phase

All the patients but one (97%) received the 3 planned courses of chemotherapy. One patient stopped the neoadjuvant chemotherapy after the second course because of grade III diarrhoea lasting more than 5 days. A single episode of grade III stomatitis was observed in one patient after the third course. A weight loss between 5% and 10% was recorded in 23% of the patients. Excluding alopecia and vomiting, other toxicities were mild to moderate (Table 3). We observed 7 CRs (23.3%), 19 PRs (63.3%), 4 SDs (13.3%) in the nasopharynx and 3 CRs (12%), 20 PRs (83%) and 1 SD (4%) in the neck. Overall, 3 patients reached a CR (10%), 25 patients a PR (83.3%) and 2 patients had a SD (6.7%).

**Table 1** Tumour and node staging

| | T1 | T2 | T3 | T4 | Total |
|---|---|---|---|---|---|
| N0 | – | – | – | 4 | 4 |
| N1 | – | – | – | 3 | 3 |
| N2a | 1 | 2 | – | 2 | 5 |
| N2b | – | 6 | – | 4 | 10 |
| N2c | – | 1 | 2 | 3 | 6 |
| N3 | 1 | – | – | 1 | 2 |
| Total | 2 | 9 | 2 | 17 | |

### Alternating phase

The chemo-radiation treatment was delivered on an out-patient basis in any case. All patients but one (97%) received the planned number of chemotherapy courses. One patient received only the first course of cisplatin and fluorouracil due to a persistent increase of the serum creatinine values (ranging between 2.5 and 3.0 mg $dl^{-1}$). All the patients received the planned radiation dose. The dose of radiation administered on the primary tumour was 70 gray in 27 patients. 3 patients with T1–2 lesions in CR after induction chemotherapy received 60 gray. The dose of radiation administered on the neck was 70 gray in 23 patients. 3 patients in CR after induction chemotherapy received 60 gray and 4 patients without nodal involvement at the time of diagnosis had 50 gray. A treatment delay of one week was needed in 5 patients (16.7%) and of 2 weeks in 2 (6.7%). The reason for the delays was correlated to the severity of local toxicity in 4 patients.

The main toxicity (Table 3) was local: 33.3% of the patients suffered from grade III–IV mucositis and despite supportive care including tube feeding and intravenous hydration given in most of the cases, these patients had a weight loss ranging between 10% and 15%. The median time to recovery to normal enteral nutrition for these patients was 4 weeks (range 1–8 weeks) from the end of the treatment. Grades II–III dermatitis was observed in 16.7% of the patients. Haematological toxicity was generally mild to moderate: 16.7% of the patient had grade III neutropenia and 10% grade III–IV thrombocytopenia.

The clinical and radiological evaluation performed 2 months after the end of the treatment programme showed 26 CRs (86.7%) and 4 PRs (13.3%). At the time of the present analysis (June 2000) the median follow-up calculated from the end of the treatment programme was 31 months (range 8–56). 9 patients had a disease progression and 1 patient developed an adenocarcinoma of the lung (Table 4). The rates of locoregional progressions and distant metastases were 13.3% and 20%, respectively.

Two out of 3 patients who developed locoregional progression only had a WHO type 2 histology, while 5/6 patients who developed distant metastases had a WHO type 3 histology. Distant metastates were detected at 7, 9, 9, 15, 22 and 58 months from the treatment beginning.

One patient out of those progressed refused any further treatment. One patient was reirradiated for a local progression and again had a CR lasting 24 months. 5 patients had a taxanes-based second-line chemotherapy and 3 of them partially responded. Two patients

**Table 2** Toxicity

| | Induction chemotherapy | | | | Chemo-radiotherapy | | | |
|---|---|---|---|---|---|---|---|---|
| WHO Grade | I (%) | II (%) | III (%) | IV (%) | I (%) | II (%) | III (%) | IV (%) |
| Leucopenia | 2 (6.7) | 3 (10) | 3 (10) | – | 3 (10) | 11 (36.7) | 5 (16.7) | – |
| Thrombocytopenia | – | – | – | – | 4 (13.3) | 2 (6.7) | 2 (6.7) | 1 (3.3) |
| Anaemia | 6 (20) | 3 (10) | – | – | 11 (36.7) | 6 (20) | 1 (3.3) | – |
| Nausea/vomiting | 2 (6.7) | 14 (46.7) | 8 (26.7) | – | 7 (23.3) | 12 (40) | 4 (13.3) | – |
| Mucositis | 5 (16.7) | 1 (3.3) | 1 (3.3) | – | 3 (10) | 14 (46.7) | 8 (26.7) | 2 (6.7) |
| Alopecia | – | 10 (33.3) | 20 (66.6) | – | – | – | – | – |
| Diarrhoea | – | – | 1 (3.3) | – | – | – | 1 (3.3) | – |
| Fever | – | 3 (10) | – | – | – | 1 (3.3) | – | – |
| Renal | – | – | – | – | 1 (3.3) | – | – | – |
| Neurotoxicity | – | – | – | – | 9 (30) | 2 (6.7) | 1 (3.3) | – |
| Dermatitis | – | – | – | – | 4 (13.3) | 2 (6.7) | 3 (10) | – |

© 2000 Cancer Research Campaign

**Table 3** Compliance with treatment

| | No. of patients | % |
|---|---|---|
| Overall no. of CT courses | | |
| 6 | 28 | 93.3 |
| 5 | 1 | 3.3 |
| 4 | 1 | 3.3 |
| Neoadjuvant CT (RDI) | | |
| 100 % | 23 | 76.7 |
| >90 % | 3 | 10 |
| ≤90 % | 4 | 13.3 |
| Alternating CT (RDI) | | |
| 100 % | 20 | 66.7 |
| >90 % | 6 | 20 |
| ≤90 % | 4 | 13.3 |
| RT dose (T) | | |
| 70 Gy | 27 | 90 |
| 60 Gy | 3 | 10 |
| RT dose (N) | | |
| 70 GY | 23 | 76.7 |
| 60 Gy | 3 | 10 |
| 50 Gy | 4 | 13.3 |

CT = chemotherapy; RT = radiotherapy; RDI = relative dose intensity; T = tumour; N = nodes

**Table 4** Incidence and site of progression

| | No. of patients | % |
|---|---|---|
| Local only | 2 | 6.7 |
| Local and nodal | 1 | 3.3 |
| Nodal and distant | 1 | 3.3 |
| Distant only | 5 | 16.7 |
| II primary | 1 | 3.3 |
| | 10 | 33.3 |
| Liver | 3 | 10 |
| Bone | 4 | 13.3 |
| Bone marrow | 1 | 3.3 |
| Skin | 1 | 3.3 |

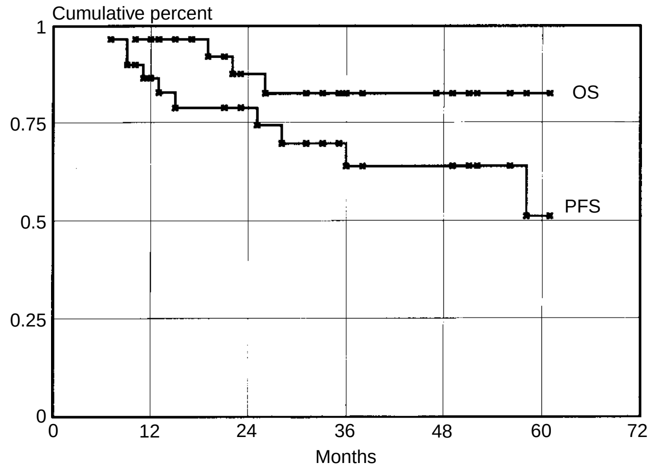

**Figure 1** Overall survival (OS) and progression free survival (PFS)

with liver metastases underwent a high-dose chemotherapy programme with peripheral stem cell support. They both reached a PR and progressed after 2 and 4 months respectively.

The 2-year actuarial progression-free survival and overall survival rates were 79% and 88%. The 3-year actuarial progression-free survival and overall survival rates were 64% and 83% (Figure 1).

### DISCUSSION

Radiotherapy alone has been considered up to now the standard treatment of locally advanced UNPC. Due to the low incidence of this disease only a few randomized trials investigating the role of chemotherapy in a front line treatment are available. The first study published (Rossi et al, 1988) failed to demonstrate any benefit for the addition of adjuvant chemotherapy after radiation. However, in this study the chemotherapy combination did not include cisplatin, which is the most active drug in this disease. A smaller trial (Chan et al, 1995) tested an experimental treatment in which chemotherapy was given before and after radiation. Also in this case no benefit was observed over radiotherapy alone. Two studies investigated the role of chemotherapy administered only before radiation. A statistically significant advantage in progression-free survival but not in overall survival was reported in one (VUMCA, 1996; El Guedari, 1998). No statistically significant benefit was observed in the other one (Chua et al, 1998). Concomitant chemo-radiotherapy followed by adjuvant chemotherapy was the experimental treatment in the Intergroup trial (Al Sarraf et al, 1998). This strategy led to a statistically significant improvement of progression-free survival and overall survival. The incidence of distant metastases was decreased from 61% to 23% and that of locoregional failures from 43% to 15% at a median follow up of 2.7 years. What we learned from these trials can be summarized as follows. The only therapeutical approach showing a clear benefit over standard radiation is, at present, that including concomitant chemo-radiotherapy. Therefore, by now, concomitant chemoradiation should be seriously considered as a part of any treatment programme for locally advanced UNPC.

The role of a sequential approach including induction and/or adjuvant chemotherapy and standard radiotherapy is still debated. If a benefit does exist it is probably small.

The addition of neoadjuvant or adjuvant chemotherapy to a concomitant chemoradiation programme like in the intergroup trial, could be a fruitful strategy in the attempt to reduce further the incidence of distant metastases and possibly locoregional relapses.

The main limit to adjuvant chemotherapy in head and neck cancer is the low patient compliance. In fact, the proportion of patients not receiving an adequate number of adjuvant chemotherapy courses after a locoregional treatment, for any cause, is generally high. On the contrary, compliance to induction chemotherapy is generally better. Moreover, an active induction regimen may be able both to reduce the tumour mass before the local treatment and to eradicate early systemic micrometastases. For these reasons in our trial we choose to give chemotherapy before the alternating regimen. To the best of our knowledge this is the first experience reported with a similar approach.

The main finding from the present study is regarding the feasibility of this combined treatment. Despite the duration of the whole programme being very long (20 weeks) if compared to that of a radiation alone or a concomitant chemo-radiation approach (7 weeks), the patients compliance was notable.

None interrupted the treatment early and 93% of the patients received all the 6 courses of chemotherapy planned in the 2 phases of the treatment. Moreover, the relative dose intensity of the drugs exceeded 90% in 87% of the patients both in the induction and in the alternating phase. These data compare favourably to those

*© 2000 Cancer Research Campaign*

reported by others with adjuvant chemotherapy. 20% of the patients never started or stopped adjuvant chemotherapy early in the Rossi trial; 46% of the patients did not receive postradiation chemotherapy as planned in the Chan trial; one third of the patients never started and only one half had the planned adjuvant chemotherapy in the Intergroup study.

Our programme had acceptable toxicity. Haematological side effects were generally mild to moderate and the incidence of severe mucositis (33%) during the alternating programme was similar to that expected with radiation alone. These data are in line with those we reported previously with alternating chemoradiation (Merlano et al, 1992). Another promising finding from our study is regarding the activity. 87% of CRs at the end of the whole treatment programme, 3-year progression-free survival rate of 64% and overall survival rate of 83% compare favourably to those reported in the literature, considering that only patients with stage IV disease were included in this trial and that most of the events generally occur during the first 3 years following treatment, and before 18 months in a high proportion of cases (Hoppe et al, 1976; Vikram et al, 1985; Chen et al, 1989; Qin et al, 1988). In fact, it should be considered that 5-year survival of patients with stage III–IV UNPC reported in uncontrolled studies with radiation alone is 15–40% (Huang, 1980; Chu et al, 1984; Zhang et al, 1987; Qin et al, 1988; Lee, 1992) and 3-year progression-free survival and overall survival rates reported in the radiation alone arm of the 2 randomized trials whose median follow-up is similar to that of our study (30–31 months) are 24% and 47% (Al Sarraf et al, 1998) and 42% and 71% (Chua et al, 1998). Even the frequencies of distant metastases and locoregional progressions seem to be lower in our study (20% and 13%, respectively) than in the Intergroup trial (61% and 43%) and in the Chua trial (25% and 30%) with radiation alone. It must be stressed, however, that in those trials also patients with stage III disease (according to the Ho's stage in the Chua trial) were enrolled and that in the Intergroup trial 25% of the patients had a WHO I histology, that means a lower metastasizing behaviour.

In conclusion, induction chemotherapy followed by an alternating chemo-radiotherapy regimen as we did in our trial showed an acceptable toxicity and an optimal patient compliance. Results in terms of activity seem to confirm the superiority of chemoradiation over radiotherapy alone shown by the Intergroup trial. If additional effective chemotherapy is needed to further improve the results of the concomitant treatment, the administration of chemotherapy before the concomitant treatment is in our opinion the more feasible option. However, a randomized trial is needed to test this hypothesis.

### REFERENCES

Al-Sarraf M, LeBlanc M, Shanker Giri PG, Fu KK, Cooper J, Vuong T, Forastiere A, Adams G, Sakr WA, Schuller DE and Ensley JF (1998) Chemoradiotherapy versus radiotherapy in patients with advanced naopharyngeal cancer: phase III randomized intergroup study 0099. *J Clin Oncol* **16**: 1310–1317

Chan ATC, Teo PML, Leung TWT, Leung SF, Lee WY, Yeo W, Choi PHK and Johnson PJ (1995) A prospective randomized study of chemotherapy adjunctive to definitive radiotherapy in advanced nasopharyngeal carcinoma. *Int J Radiat Oncol Biol Phys* **33**: 569–577

Chatani M, Teshima T, Inoue T, Azuma I, Yoshimura H, Oshitani T, Hashiba M, Nishiyama K, Tsutsui K and Fujimura T (1986) Radiation therapy for nasopharyngeal carcinoma. Retrospective review of 105 patients based on a survey of Kansay Cancer Therapist Group. *Cancer* **57**: 2267–2271

Chen WZ, Zhou DL and Luo KS (1989) Long term observation after radiotherapy for nasopharyngeal carcinoma (NPC). *Int J Radiat Oncol Biol Phys* **16**: 311–314

Chu A, Flynn MB, Achino E, Mendoza EF, Scott RM and Jose B (1984) Irradiation of nasopharyngeal carcinoma: correlations with treatment factors and stage. *Int J Rad Oncol Biol Phys* **10**: 2241–2249

Chua DTT, Sham JST, Choy D, Lorvidhaya V, Sumitsawan Y, Thongprasert S, Vootiprux V, Cheirsilpa A, Azhar T and Reksodiputro AH (1998) Preliminary report of the Asian-Oceanian Clinical Oncology Association randomized trial comparing cisplatin and epirubicin followed by radiotherapy versus radiotherapy alone in the treatment of patients with locoregionally advanced nasopharyngeal carcinoma. *Cancer* **83**: 2270–2283

Cvitkovic E (1991) Nasopharyngeal carcinoma: biology, natural history and therapeutic implications. *Hemat Oncol Clinics North America* **5**: 821–838

Dexing Q, Yuhua H and Jiehua J (1988) Analysis of 1379 patients with nasopharyngeal carcinoma treated by radiation. *Cancer* **61**: 1117–1124

El Guedari B (1998) Final results of the VUMCA I randomized trial comparing neoadjuvant chemotherapy (CT) (BEC) plus radiotherapy (RT) to RT alone in undifferentiated nasopharyngeal carcinoma (UCNT). *Proc Am Soc Clin Oncol* **17**: 385 (abstr 1482)

Ho JHC (1978) An epidemiologic and clinical study of nasopharyngeal carcinoma. *Int J Radiat Oncol Biol Phys* **4**: 183–188

Ho JHC (1982) Cancer in Hong Kong: some epidemiological observations. *NCI Monogr* **62**: 47–55

Hoppe RT, Goffinet DR and Bagshaw MA (1976) Carcinoma of the nasopharynx. Eighteen year's experience with megavoltage radiation therapy. *Cancer* **37**: 2605–2612

Huang SC (1980) Nasopharyngeal cancer: a review of 1605 patients treated with Cobalt 60. *Int J Rad Oncol Biol Phys* **6**: 401–407

Huang SC, Lui LT and Lynn TC (1985) Nasopharyngeal cancer: study III. A review of 1206 patients treated with combined modalities. *Int J Rad Oncol Biol Phys* **11**: 1789–1793

Kaplan EL and Meier P (1958) Nonparametric estimation for incomplete observations. *J Am Stat Assoc* **53**: 457–481

International Nasopharynx Cancer Study Group (VUMCA 1) (1996) Preliminary results of a randomized trial comparing neoadjuvant chemotherapy (cisplatin, epirubicin, bleomycin) plus radiotherapy vs radiotherapy alone in stage VI (≥N2,M0) undifferentiated nasopharyngeal carcinoma: A positive effect on progression-free survival. *Int J Radiat Oncol Biol Phys* **35**: 463–469

Lee AWM (1992) Retrospective analysis of 5037 patients with nasopharyngeal carcinoma treated during 1976–1985: overall survival and patterns of failure. *Int J Radiat Oncol Biol Phys* **23**: 261–270

Levine PH and Conelly RR (1985) Epidemiology of nasopharyngeal cancer. In: Wittes R (ed), *Head and Neck Cancer*. J Wiley, Chichester, pp. 13–34

Li CC (1985) Some epidemiologic observations of nasopharyngeal carcinoma in Guandong People's Republic of China. *NCI Monogr* **69**: 49–52

Licitra L, Grandi C, Cavina R, Zucali R, Guzzo M, Demicheli R and Molinari R Bonadonna G (1994) Primary chemotherapy with cisplatin and epirubicin plus radiotherapy for advanced nasopharyngeal carcinomas (NPC). *Head & Neck* **16**: 491

Merlano M, Vitale V, Rosso R, Benasso M, Corvo' R, Cavallari M and Sanguineti G (1992) Treatment of advanced squamous cell carcinoma of the head and neck with alternating chemotherapy and radiotherapy. *N Engl J Med* **327**: 1115–1121

Mesic JB, Fletcher GH and Goepfert H (1981) Megavoltage irradiation of epithelial tumors of the nasopharynx. *Int J Radiat Oncol Biol Phys* **7**: 447–453

Micheau C, De The G and Orofiamma B (1981) Practical value of classifying NPC in two major microscopical types. In: Grundmann et al. (eds) *Cancer Campaign, Vol 5, Nasopharyngeal Carcinoma*. Gustav Fischer Verlag, Stuttgart, New York

Miller AB, Hoogstraten B, Staquet M and Winkler A (1981) Reporting results of cancer treatment. *Cancer* **71**: 485–488

Oliveira J, Martins A, Lage P, Gil N, Magalhaes M and Araujo F (1996) Cisplatin and high-dose epirubicin in non-queratinizing epidermoid carcinoma of the nasopharynx. *Proc Am Soc Clin Oncol* **15**: 313 (abstr 880)

Qin D, Hu YH, Yan JH, Xu GZ, Cai WM, Wu XL, Cao DX and Gu XZ (1988) Analysis of 1379 patients with nasopharyngeal carcinoma treated by radiation. *Cancer* **61**: 1117–1124

Rahal M, Djemaa A, Filali K and Boudaoud K (1994) Preliminary report of high dose epirubicin and cisplatin induction chemotherapy in locally advanced bulky undifferentiated carcinoma of nasopharingeal type. *Ann Oncol* **5** (Suppl 8): 159 (abstr P593)

© 2000 Cancer Research Campaign

Rossi A, Molinari R, Boracchi P, Del Vecchio M, Marubini E, Nava M, Morandi L and Zucali R (1988) Adjuvant chemotherapy with Vincristine, Cyclophosphamide, and Doxorubicine after radiotherapy in local-regional nasopharyngeal cancer: results of a 4-year multicenter randomized study. *J Clin Oncol* **6**: 1401–1410

Sanguineti G, Geara FB, Gargen AS, Tucker SL, Ang KK, Morrison WH and Peters LJ (1997) Carcinoma of the nasopharynx treated by radiotherapy alone: determinants of local and regional control. *Int J Radiat Oncol Biol Phys* **5**: 985–996

Shwaab G (1983) Les carcinomes du nasopharynx: étude anatomopathologique, clinique, traitements, resultats. In: Lemerle J (ed) *Act Carcinologiques*. Paris: Masson, pp. 88–89

Vikram B, Mishra UB, Strong EW and Manolatos S (1985) Patterns of failure in carcinoma of the nasopharynx: failure at the primary site. *Int J Radiat Oncol Biol Phys* **11**: 1455–1459

Zhang EP, Liang PG and Li ZQ (1987) Ten-year survival of nasopharyngeal carcinoma. A report of 1302 cases. *Chin Med J* **100**: 419–424

 *© 2000 Cancer Research Campaign*
